# Visualisation and network analysis of physical activity and its determinants: Demonstrating opportunities in analysing baseline associations in the Let’s Move It trial

**DOI:** 10.1080/21642850.2019.1646136

**Published:** 2019-07-25

**Authors:** Matti T. J. Heino, Keegan Knittle, Eiko Fried, Reijo Sund, Ari Haukkala, Katja Borodulin, Antti Uutela, Vera Araujo-Soares, Tommi Vasankari, Nelli Hankonen

**Affiliations:** a Faculty of Social Sciences, University of Helsinki Helsinki, Finland; b Department of Clinical Psychology, Leiden University, Leiden, The Netherlands; c Faculty of Health Sciences, University of Eastern Finland Kuopio, Finland; d Institute of Health and Society, Medical Faculty, Newcastle University, Newcastle upon Tyne, UK; e UKK institute for Health Promotion Research Tampere, Finland

**Keywords:** Exercise, physical activity, school-based intervention, behaviour change, sedentary behaviour

## Abstract

**Background:** Visualisations and readily-accessible web-based supplementary files can improve data reporting and transparency. In this paper, we make use of recent developments in software and psychological network analysis to describe the baseline cohort of a trial testing the Let’s Move It intervention, which aimed to increase physical activity (PA) and reduce sedentary behaviours (SB) among vocational school students.

**Methods:** At baseline, 1166 adolescents, distributed across 6 school clusters and four educational tracks, completed measures of PA and SB, theoretical predictors of these behaviours, and body composition. Within a comprehensive website supplement, which includes all code and analyses, data were tabulated and visualised, and network analyses explored relations between predictor variables and outcomes.

**Results:** Average daily moderate-to-vigorous PA was 65 min (CI95: 57min–73 min), and SB 8h44 min (CI95: 8h04min–9h24 min), with 25.8 (CI95: 23.5–28.0) interruptions to sitting. Cluster randomisation appeared to result in balanced distributions for baseline characteristics between intervention and control groups, but differences emerged across the four educational tracks. Self-reported behaviour change technique (BCT) use was low for many but not all techniques. A network analysis revealed direct relationships between PA and behavioural experiments, planning and autonomous motivation, and several BCTs were connected to PA via autonomous motivation. Visualisation uncovered a case of Simpson’s paradox.

**Conclusions:** Data-visualisation and data exploration techniques (e.g. network analysis) can help reveal the dynamics involved in complex multi-causal systems – a challenging task with traditional data presentations. The benefits of presenting complex data visually should encourage researchers to publish extensive analyses and descriptions as website supplements, which would increase the speed and quality of scientific communication, as well as help to address the crisis of reduced confidence in research findings. We hope that this example will serve as a template for other investigators to improve upon in the future.

## Background

Declining physical activity (PA) and increasing sedentary behaviour (SB) are costly and growing concerns for public health, especially among individuals with low socioeconomic status (SES) (Dieleman et al., 2018; Elgar et al., 2015). Patterns of low PA among adults begin earlier in the life course, with evidence that declines in PA and increases in SB begin during childhood and adolescence (Husu, Vähä-Ypyä, & Vasankari, 2016; Mäkelä et al., 2016). This highlights the need for further research into interventions to improve PA and SB among adolescents.

As adolescents spend a significant amount of their time in schools, the school setting provides valuable opportunities for PA and SB interventions (van Sluijs et al., 2008). The Let’s Move It intervention aimed to reduce SB and increase PA among adolescents in vocational schools, and was developed using stakeholder input and co-creation with target group representatives, as well as theories and empirical evidence from behavioural science (Hankonen, Heino, Kujala, et al., 2017; Hynynen et al., 2016). Contrary to typical school-based interventions with relatively homogeneous participants, this trial was carried out in vocational schools with distinct and varied educational tracks (i.e. practical nurse, business information and communication technology, business administration, and hotel, restaurant and catering). Understanding the implications of these distinct tracks on the way participants engage in both PA and SB will support a better understanding of the individual and contextual determinants of behaviour and more informed interpretations of the results obtained in the trial.

The hypothesised programme theories (Moore et al., 2015; Rogers, 2008) for changing PA and SB differed from one another. In order to increase PA, one needs to make a conscious effort and implement self-regulatory skills (e.g. action planning and overcoming barriers to PA) to make optimal use of opportunities. The Let’s Move it intervention places a particular emphasis on helping adolescents understand and use techniques to manage their motivation and behaviour (see also Hankonen, Heino, Hynynen, et al. (2017) and Hankonen (2018)). To date, there is little knowledge about how the use of these techniques links to each other, and it would be important to examine these links empirically. The theoretical model for changing SB, on the other hand, is more driven by environmental opportunities, such as having the option to stand up during class.

In order to increase moderate-to-vigorous-intensity PA, the Let’s Move It intervention targeted several behavioural determinants, including behavioural beliefs (outcome expectations, descriptive norms, intention, self-efficacy/perceived behavioural control), autonomous and controlled motivation, environmental opportunities, action and coping planning, and behaviour change technique (BCT) use. Key hypotheses regarding students’ PA change have been registered in OSF (https://osf.io/tb8fu/). To reduce total SB and introduce breaks in SB, the programme aimed to change the school environment by training teachers in the use of active teaching techniques and altering physical choice architecture in classrooms (Köykkä et al., 2018). The intervention also included poster campaigns in schools, a website, and materials to target community actors and parents (Köykkä et al., 2018). More information of the content of the intervention and the development of it is reported elsewhere (Hankonen et al., 2016; Hankonen, Absetz, & Araujo-Soares, 2019; Hankonen, Heino, Hynynen, et al., 2017).

It has long been a standard recommendation for quantitative analyses to investigate data visually as a core precursor of conducting statistical analyses (Cleveland, 1993; Tukey, 1977). However, in social and life sciences, such visualisations are rarely shared in publications. Information about data are usually limited to means and standard deviations, which presents at best limited information about the variables of interest (Trafimow, Wang, & Wang, 2018). Medians, modes, skewness and kurtosis provide helpful additional information, but human cognition places limits on evaluating these statistics simultaneously, especially when comparing groups of observations. For example, two distributions can have different means but the same mode, different modes but the same mean, or the same mean and standard deviation but a meaningful skew. Summary statistics conventionally calculated from the data leave important distributional properties uncovered, as illustrated in recent discussions on the inadequacy of bar plots (Saxon, 2015; Weissgerber, Garovic, Savic, Winham, & Milic, 2016; Weissgerber, Milic, Winham, & Garovic, 2015).

Data visualisations are crucial supplements to large numerical tables of descriptive statistics (Tay, Parrigon, Huang, & LeBreton, 2016). With visualisations, researchers can communicate large amounts of information – including the associated uncertainty – in an accessible format, without requiring extensive mathematical expertise from the reader. This is important for researchers who intend to build on previous results (Chalmers & Glasziou, 2009). Such practices may reduce problems that have led to the recent loss of confidence in the reproducibility and replicability of research findings (Gigerenzer, 2018; Kepes & McDaniel, 2013; Nosek, Ebersole, DeHaven, & Mellor, 2018; Nosek, Spies, & Motyl, 2012; Simmons, Nelson, & Simonsohn, 2011; Smaldino & McElreath, 2016). Fully open data sharing would be ideal, but this is not always possible due to privacy concerns (Expert Advisory Group on Data Access, 2015) and, at the time of writing, remains a lamentably rare practice (Vanpaemel, Vermorgen, Deriemaecker, & Storms, 2015). In addition, open data does not necessarily accommodate stakeholders with low technical expertise in data analysis and visualisation, such as clinicians, patients and policy makers; see Hallgren, McCabe, King, and Atkins (2018), p. 2.

Three recent developments give impetus to a new approach. First, many journals now allow publication of supplementary online materials, which circumvents both word and figure restrictions of traditional manuscripts. Second, statistical software such as R (R Core Team, 2015) has recently become increasingly mainstream among applied researchers, with many free tutorials available online, opening the door for a variety of data visualisation techniques. Third, novel statistical methods in social and health psychology, such as psychological network analysis, may help to understand relationships between variables by making better use of visual representations of associations.

The aims of this paper are to describe central characteristics of the Let’s Move It trial baseline cohort, focusing on co-primary outcomes and other activity measures (as measured by accelerometry) of the trial both arms, genders and educational tracks in both trial arms. A further aim is to describe psychological and social correlates, as well as hypothesised determinants of the intervention’s effect on moderate-to-vigorous PA (MVPA), with detailed visualisations of the dataset provided in an extensive supplementary website. As a sub-aim, we also investigate the network of relationships between MVPA, quality of motivation and BCT use at baseline. We provide all code as open source scripts, so that other researchers can use those scripts as templates to visualise their own datasets in a format that requires no special skills or tools to view.

## Methods

This study analyses baseline data from a cluster-randomised controlled trial testing Let’s Move It, a complex whole-school system multi-level intervention conducted in Finnish vocational schools. Details of the Let’s Move It trial have been described in the study protocol (Hankonen et al., 2016). At baseline, consenting participants in both intervention and control groups answered an electronic survey, underwent bioimpedance measurements and were instructed to wear an accelerometer for seven consecutive days. The baseline data collection started in January 2015 and ended in April 2016.

Six school units were included in the study. There were four educational tracks in the schools from which students were recruited: 1. Practical Nurse (Nur), 2. Hotel, Restaurant and Catering (HRC), 3. Business and Administration (BA), and 4. Information and Communications Technology (IT). Schools were paired so that there would be matching numbers of students from each educational track for both members of the pair. Blinded randomisation by a statistician was then conducted so that a random member of each pair was selected as intervention school, the other as control school (details reported in Hankonen et al. (2016)). Student participants provided informed consent and were blind to allocation at baseline.

All conducted analyses and visualisations with accompanying code, can be found in the supplementary website at https://git.io/fNHuf (permalink at Heino and Sund (2019)), previously piloted in (Heino, Knittle, Haukkala, Vasankari, & Hankonen, 2018). Source code to reproduce this manuscript (written with the R package papaja (Aust & Barth, 2019)), and all its figures can be found at https://git.io/fptcC.

### Measures

The measures are presented briefly, as they have been previously described in Hankonen et al. (2016), and all individual items of the scales are available in the supplementary website (see section https://git.io/fjfLw).

#### Primary outcome variables of the trial

In the LMI trial, there were multiple primary outcomes. The primary outcome for PA was moderate to vigorous PA (MVPA), measured by accelerometry and self-reports. Primary outcomes for sedentary behaviour (SB) were measured by accelerometry; they included time spent sitting or lying down, and the number of times sitting was interrupted during the day.

##### Self-reported MVPA

Self-reported MVPA was measured with two questions in accordance with the NordPAQ measurement (Fagt et al., 2012). The first question asked participants about the number of days during the last week in which they did more than 30 min of MVPA, the other probed the overall amount of MVPA (in hours) during the past seven days.

##### Accelerometer-measured MVPA and SB

No more than seven days after responding to the questionnaire, students were given an accelerometer to be worn on seven consecutive days. The hip-worn accelerometer (Hookie AM 20, Traxmeet Ltd, Espoo, Finland) using a digital triaxial acceleration sensor (ADXL345; Analog Devices, Norwood MA) was attached to a flexible belt and participants were instructed to wear the belt around their right hip for seven consecutive days during waking hours, except during shower and other water activities. The acceleration signal was collected at 100 Hz sampling frequency, ±16 g acceleration range and 0.004 g resolution. Definitions of the parameters are described in detail in the supplementary website (section https://git.io/fjJNi).

#### Theoretical predictors of PA

The determinants postulated by the programme theory included behavioural beliefs (outcome expectations, descriptive norms, intention, self-efficacy/perceived behavioural control), autonomous and controlled motivation, opportunities, action- and coping planning, and behaviour change technique (BCT) use. Participants were allowed to skip questions, and scales were computed as means of all items where responses were available. In other words, answering a single item of a specific scale sufficed. For the scales, all items, response options, descriptive statistics, as well as information about missing values and estimated reliability coefficients, are available in the supplementary website (section https://git.io/fAj0e); made using R package codebook (Arslan, in press) for automatic dataset documentation.

### Statistical analysis

We used RStudio (RStudio Team, 2015) 1.1.456 running R (Version 3.6.0; R Core Team, 2018) for all our analyses and figures.

In our case (no confirmatory hypotheses), confidence intervals are more appropriate to report than *p*-values, as they provide readily interpretable values on the same scale as the original variable, accommodating inferences of practical relevance (Gardner & Altman, 1986; Nosek et al., 2018; Sterne, 2001; Wasserstein & Lazar, 2016). Hence, we omit explicit statistical testing from the tables.

Activity data was explored by utilising 100% stacked bar charts, which are useful when comparing proportions which add to 100%. MVPA data was, in addition, examined with augmented raincloud ridge plots to unveil distributional properties. Psychological and social determinants were examined with diamond plots (Peters, 2018), and heuristic (here: not taking into account the clustering of the participants into schools and classrooms) effect sizes between means of intervention arms and genders, transformed from Cohen’s d to Pearson’s r.

Psychological network analysis was used to estimate and visualise relations among BCT use, motivation and MVPA. Such networks contain nodes (variables) and edges (statistical relationships between variables). Unlike in social network analysis, the connections are not directly observed, but are estimated. We used network models that estimate conditional dependence relations among a set of variables, which can be interpreted similarly to partial correlations. An edge between two variables implies that they are related after controlling for all other variables; the absence of an edge implies that the two variables are (conditionally) independent.

The Mixed Graphical Model uses regularisation, a procedure that has been shown to help recover the true network structure in data in case the data were simulated under a network model (Haslbeck & Waldorp, 2015). Regularisation has the goal to avoid estimating spurious relationships among items (i.e. false positive relations), and results in a parsimonious network structure. The regularisation technique used here is the Least Absolute Shrinkage and Selection Operator (LASSO; Tibshirani (2011)), which shrinks all edges and sets very small edges to exact zero. A paper that explains LASSO regularisation in network models in detail can be found elsewhere (Epskamp & Fried, 2018).

Network models applied to between-subjects data at one time-point can be useful for describing health psychological data, as well as facilitating group-level hypothesis generation regarding which parts of the system are central for a problem at hand (Fried & Cramer, 2017). Identifying these determinants of importance can thus supplement traditional structural equation modeling (SEM) approaches. SEM usually specifies *directed* models, usually in an acyclic manner (i.e. disregarding feedback loops). This can be valuable for confirmatory modelling in multivariate data when there has been previous work on understanding putative causal effects of the involved variables. However, due to model equivalence—the fact that often many dozen of undirected path models can be fit to the same data with identical fit (Stelzl, 1986)—directed models can be challenging to use in highly multivariate, exploratory cases. All of these equivalent directed models can be subsumed into one undirected model, a network model that estimates and visualises the multivariate conditional dependence relations highly relevant in health psychological contexts, where many causal factors contribute to produce effects in a mutually reinforcing manner.

Network analysis has recently been taken up in many fields such as social psychology (Dalege, Borsboom, van Harreveld, Waldorp, & van der Maas, 2017, 2016), personality (Mõttus & Allerhand, 2017), intelligence (Van Der Maas, Kan, Marsman, & Stevenson, 2017), psychopathology (Fried et al., 2017), and empathy research (Briganti, Kempenaers, Braun, Fried, & Linkowski, 2018), and is beginning to be applied for health behaviours on a broader scale. Several helpful tutorial papers aimed at empirical researchers are available (Costantini et al., 2015, 2019; Dalege, Borsboom, van Harreveld, & van der Maas, 2017; Epskamp & Fried, 2018; Epskamp, Borsboom, & Fried, 2018), and also exist for health psychology context in particular (Hevey, 2018).

To ease interpretation of the network analysis, we dichotomised the heavily skewed controlled motivation variable in such a way that 1 represents answers 3 (‘partly true for me’) or higher, and 0 the rest. In addition, BCT use variables were dichotomised by giving 0 if a person reports completely disagreeing with their statements, or never having used the technique, and 1 otherwise. A correlation matrix of the variables can be found in the supplement (https://git.io/fhAgk).

## Findings

In this section, we first present data in traditional numeric tables, and follow up by augmenting them with graphical illustrations. Table 1 shows the main demographic variables of the cohort by educational track. Among 638 intervention arm participants, 80.5% (429/533) reported having been born in Finland. Among the 528 control arm participants, the percentage was 88.7% (423/477).

**Table 1.** Baseline demographics of educational tracks. Omitted are 24 participants, who reported ‘other’ as their track, as well as 81 participants from whom data is not available. Nur = Practical nurse, HRC = Hotel, restaurant and catering studies, BA = Business and administration, IT = Business information technology.

Variable	Nur	HRC	BA	IT	Full sample
*n*	402	213	282	163	1166
Mean study year (sd, median)	1.7 (0.9, 1.0)	1.9 (0.7, 2.0)	1.7 (0.9, 1.0)	1.7 (0.9, 1.0)	1.7 (0.9, 1.0)
Mean age (range, median)	18.8 (16.0–49.0, 17.0)	18.5 (17.0–27.0, 18.0)	18.0 (16.0–35.0, 17.0)	18.5 (17.0–43.0, 17.0)	18.5 (16.0–49.0, 18.0)
Born in Finland (%)	80.1	88.3	89.7	86.7	84.4
% girl	82.3	60.6	39.0	16.0	56.5
% allocated to intervention	68.9	31.5	53.5	46.6	54.7

While on average the sample was relatively balanced on boys and girls (43.5% vs. 56.5%), educational tracks were heavily divided by gender: Practical Nurse track had the highest amount of girls (82.3%) and IT track lowest (16.0%). Age ranged from 16 to 49, with the average age being 18.50. Altogether there were 190 (16%) students who reported being at least 20 years old.

Table 2 shows summary statistics for primary outcome variables with their intra-class correlations (ICCs) for class and school (see supplementary website, section https://git.io/fjIcc, for ICCs of all variables). The ICC can be interpreted as the proportion of the variable’s variance accounted for by group membership.

**Table 2.** Key variables with their class and school intra-class correlations (ICCs). Let’s Move It trial’s primary outcome variables marked with asterisks. Accelerometry data is missing from 435 participants, of whom 169 due to not meeting the cutoff of at least 10 h of measurement time for at least four days. Survey data missing from 84 participants.

Variable	Mean	CI95	ICC class	ICC school	*n*
Daily moderate-to-vigorous PA time (accelerometer)*	1 h 5 min	0 h 57 min–1 h 13 min	.089	.062	731
Daily light PA time (accelerometer)	2 h 51 min	2 h 32 min–3 h 9 min	.111	.110	731
Daily standing time (accelerometer)	1 h 24 min	1 h 15 min–1 h 34 min	.122	.041	731
Daily time spent sitting or lying down (accelerometer)*	8 h 44 min	8 h 4 min–9 h 24 min	.115	.138	731
Daily number of times sitting was interrupted (accelerometer)*	25.8	23.5–28.0	.047	.080	731
Number of days with > 30 MVPA min previous week (self-report)*	2.8	2.6–3.0	.047	<.001	1082

At baseline, 63.6% students provided at least 4 days with a minimum of 10 h per day of valid accelerometer data. On average, the participants reported engaging in at least 30 min of MVPA on 2.80 days a week. Accelerometer data indicated, that girls were as active as boys (mean 65 vs. 67 min). Given that boys are generally more active than girls (Husu, Vähä-Ypyä, et al., 2016), this result will be elaborated on below.

**Figure 1.**
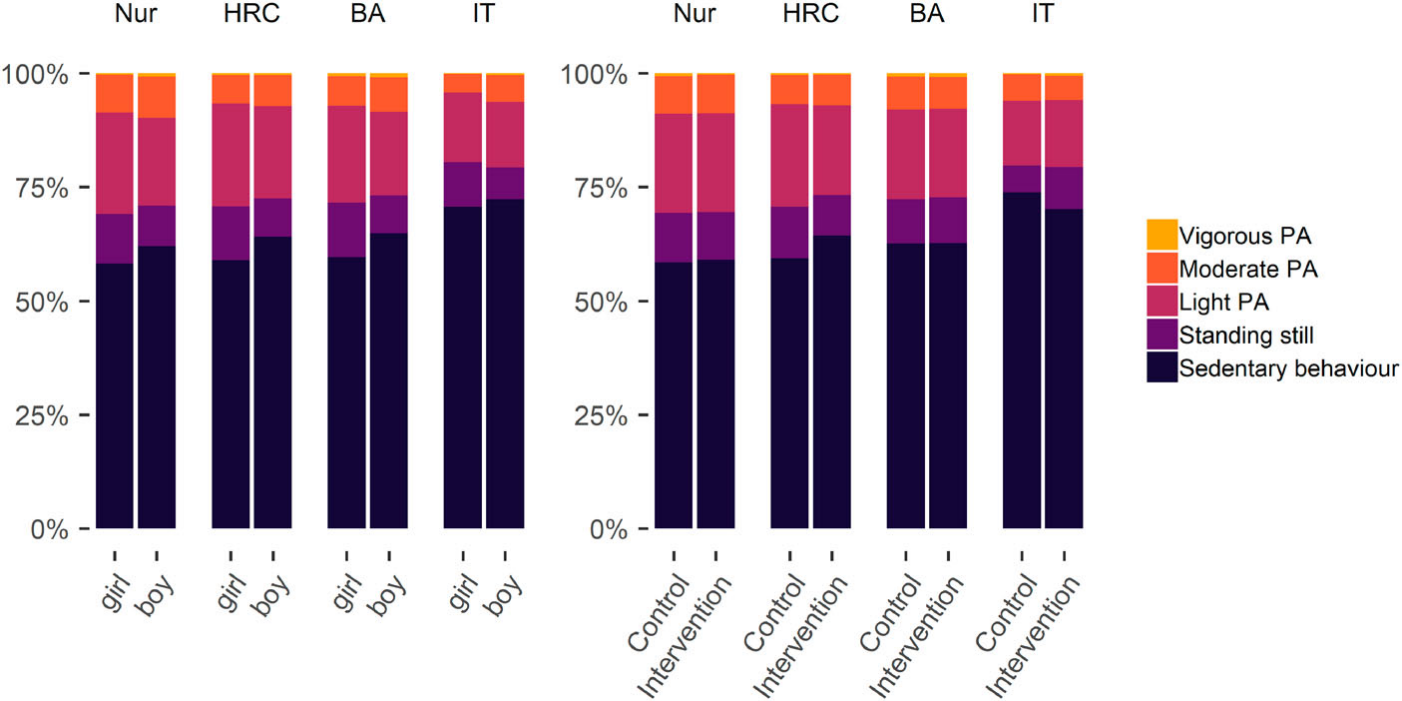
Stacked bar plot drawn with R package ggplot (Wickham et al. (2018), code available at https://git.io/fptlp), showing proportions of accelerometer-measured physical activity (PA) in relation to measurement time, averaged over genders, arms and educational tracks. Nur = Practical nurse, HRC = Hotel, restaurant and catering, BA = Business and administration, IT = Information and communications technology.

To give the reader a richer perspective than from what can be gauged from considering these summary statistics only, we present the results graphically in Figure 1. We can see that the patterns of average baseline activity, as measured by the accelerometer, are similar within gender and intervention allocation groups. However, the charts reveal that the IT track is more sedentary compared to other tracks and that girls are actually *less* active in each educational track.

The plot shows the average activity types relative to measurement time, but hides variability around the averages. The graph does not depict, for example, that while the average portion of time spent in sedentary behaviour for the IT track was 72.0%, almost half (42.0%) of that track’s participants were sedentary more than 75% of the time.

Zooming in on accelerometer-measured MVPA, Table 3 gives us statistics – some of which more commonly reported, others less so – on the variable.

**Table 3.** Statistics describing accelerometer-measured moderate-to-vigorous physical activity in different educational tracks. Values not corrected for effects of clustering.

Gender	Arm	Nur	HRC	BA	IT
girl	control	M = 73.0; SD = 29.5; skewness = 0.9; kurtosis = 0.6; *n* = 104	M = 57.5; SD = 22.3; skewness = 0.8; kurtosis = 1.0; *n* = 90	M = 61.2; SD = 23.8; skewness = 0.8; kurtosis = 1.0; *n* = 53	M = 34.2; SD = 8.9; skewness = 0.4; kurtosis=−0.8; *n* = 14
girl	intervention	M = 71.8; SD = 28.4; skewness = 1.0; kurtosis = 1.6; *n* = 227	M = 52.0; SD = 23.6; skewness = 0.8; kurtosis=−0.2; *n* = 38	M = 58.7; SD = 22.8; skewness = 1.2; kurtosis = 0.7; *n* = 58	M = 36.1; SD = 22.1; skewness = 0.2; kurtosis=−0.8; *n* = 12
boy	control	M = 72.7; SD = 28.9; skewness = 0.3; kurtosis=−1.1; *n* = 21	M = 56.1; SD = 27.4; skewness = 1.5; kurtosis = 2.0; *n* = 55	M = 70.9; SD = 27.9; skewness = 0.7; kurtosis = 0.5; *n* = 72	M = 55.8; SD = 24.7; skewness = 1.1; kurtosis = 2.7; *n* = 80
boy	intervention	M = 89.6; SD = 39.1; skewness = 0.7; kurtosis = 0.2; i = 50	M = 71.2; SD = 43.9; skewness = 0.9; kurtosis = 0.5; *n* = 28	M = 72.8; SD = 31.0; skewness = 0.4; kurtosis=−0.8; *n* = 93	M = 54.1; SD = 25.1; skewness = 1.3; kurtosis = 1.5; *n* = 65

**Figure 2.**
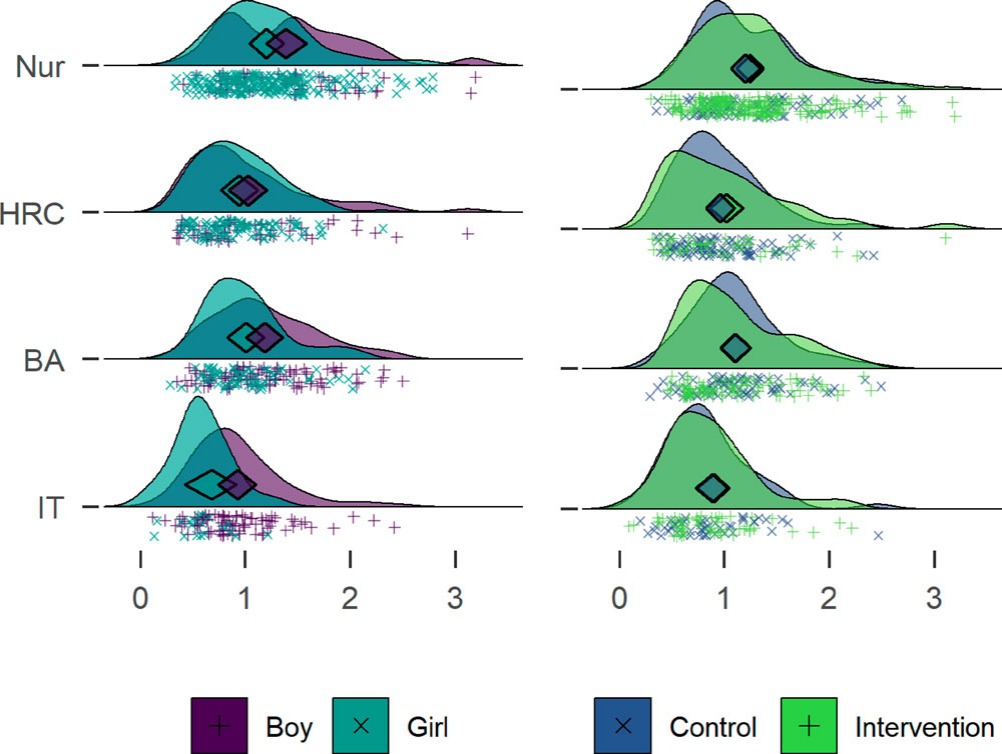
Raincloud ridge plot combined with a diamond plot, drawn with R packages ggridges (Wilke & ggridges, 2018) and userfriendlyscience (Peters, Verboon, and Green (2018), code available at https://git.io/fjLBG), showing hours of accelerometer-measured moderate-to-vigorous physical activity for different educational tracks. Midpoints of diamonds indicate means, endpoints 95% credible intervals (see (Heino, Vuorre, & Hankonen, 2018) for interpretation). Individual observations are presented under the density curves, with random scatter on the y-axis to ease inspection. Nur = Practical nurse, HRC = Hotel, restaurant and catering, BA = Business and administration, IT = Information and communications technology.

Figure 2 displays an augmented density plot, representing and elaborating on information from Table 3. The density curves can be read like a histogram, but the shape is not dependent on the bar width. They also help illustrate differences across groups, revealing potential differences in variability and distribution shape. The plot shown presents raw data below the density curve, to allow the reader to see the data on which the density algorithm is based upon. Augmenting the graph with the diamond facilitates inferences based on location of the mean. (Peters, 2018)

As the diamonds in Figure 2 illustrate, participants who study practical nursing are the most active, followed by HRC students and BA students, with the IT track being the least active. There is considerable variation within tracks though. This explains the gender difference in MVPA: the practical nurse track is the largest, and its students, mostly girls, are the most active. The IT students, mostly boys, are the least active.

In sum, boys did more MVPA in every educational track (mean differences in minutes: 12.80 for Practical nurse, 5.40 for Hotel, restaurant and catering, 11.90 for Business and administration, and 19.90 for IT). In spite of this, girls appear more active in the aggregate. This is also known as the Simpson’s paradox, and is best investigated by visualising data (see Kievit, Frankenhuis, Waldorp, and Borsboom (2013) for an introduction). Examining the left side of Figure 2 reveals the difference between boys and girls in MVPA, the difference between Practical nurse and IT tracks, the differences in gender composition, and differences in the amount of participants in each track. These, when taken together, contribute to a comprehensive understanding of the data.

Similar plots for all primary outcome variables can be found in the supplement. In brief, regardless of track, boys reported more days with at least 30 min of MVPA, while reporting more e.g. gym training, which was more strongly connected to the self-reported MVPA than the accelerometer-measured one. Accelerometer measurement also indicated, that boys engaged in more sedentary time and interrupted sitting less often than girls (see supplementary website, sections https://git.io/fjvWv and https://git.io/fjvCj).

### Theoretical determinants

In Table 4 below, we present the means for the primary outcome variables by gender and trial arm.

**Table 4.** Main theoretical determinants of physical activity (PA) and sedentary behaviour (SB). Mean (CI95, taking into account school and class membership). Action and coping planning are evaluated on a scale from 1 to 4, autonomous / controlled regulation, amotivation and behaviour change technique (BCT) use on a scale from 1 to 6 – all other variables from 1 to 7.

Variable	Girls (*n* = 603–611)	Boys (*n* = 459–467)	Intervention (*n* = 570–579)	Control (*n* = 492–499)	Total (*n* = 1062–1078)
PA intention	5.3 (5.1–5.5)	5.5 (5.2–5.7)	5.4 (5.1–5.7)	5.4 (5.1–5.7)	5.4 (5.2–5.6)
PA perceived behavioural control	5.2 (5.1–5.3)	5.5 (5.4–5.6)	5.3 (5.1–5.5)	5.3 (5.1–5.5)	5.3 (5.2–5.5)
PA self-efficacy	5.1 (5.0–5.3)	5.3 (5.2–5.5)	5.2 (5.0–5.3)	5.3 (5.1–5.4)	5.2 (5.1–5.4)
PA opportunities	5.1 (5.0–5.1)	5.2 (5.1–5.3)	5.1 (5.0–5.2)	5.2 (5.1–5.3)	5.1 (5.1–5.2)
PA descriptive norm	4.3 (4.1–4.5)	4.6 (4.4–4.7)	4.3 (4.1–4.5)	4.5 (4.3–4.7)	4.4 (4.2–4.6)
PA injunctive norm	4.6 (4.4–4.8)	4.8 (4.5–5.0)	4.5 (4.3–4.7)	4.8 (4.6–5.0)	4.7 (4.5–4.8)
PA outcome expectations	5.4 (5.2–5.5)	5.1 (5.0–5.3)	5.2 (5.0–5.5)	5.3 (5.1–5.5)	5.3 (5.1–5.4)
PA action planning	2.7 (2.6–2.8)	2.8 (2.7–2.9)	2.7 (2.6–2.8)	2.8 (2.7–2.9)	2.8 (2.7–2.8)
PA coping planning	2.4 (2.4–2.5)	2.6 (2.5–2.7)	2.5 (2.4–2.6)	2.5 (2.4–2.6)	2.5 (2.4–2.6)
PA autonomous regulation	3.3 (3.2–3.5)	3.6 (3.4–3.7)	3.4 (3.2–3.5)	3.5 (3.3–3.6)	3.4 (3.3–3.5)
PA controlled regulation	1.9 (1.8–2.0)	1.8 (1.7–1.8)	1.8 (1.7–1.9)	1.9 (1.8–1.9)	1.8 (1.8–1.9)
PA amotivation	1.5 (1.4–1.5)	1.6 (1.5–1.7)	1.5 (1.4–1.6)	1.5 (1.4–1.6)	1.5 (1.5–1.6)
PA agreement-BCTs	3.1 (2.9–3.2)	3.2 (3.0–3.3)	3.0 (2.9–3.2)	3.2 (3.0–3.4)	3.1 (3.0–3.2)
PA frequency-BCTs	2.5 (2.4–2.6)	2.6 (2.4–2.7)	2.5 (2.4–2.6)	2.6 (2.4–2.7)	2.5 (2.4–2.6)
SB intention	3.8 (3.5–4.1)	3.6 (3.3–3.9)	3.7 (3.2–4.2)	3.7 (3.3–4.2)	3.7 (3.4–4.1)
SB descriptive norm	3.2 (3.0–3.4)	3.4 (3.1–3.6)	3.2 (3.0–3.4)	3.3 (3.1–3.5)	3.2 (3.1–3.4)
SB injunctive norm	4.0 (3.8–4.1)	4.1 (3.9–4.3)	3.9 (3.8–4.1)	4.1 (4.0–4.2)	4.0 (3.9–4.1)
SB outcome expectations	4.9 (4.8–5.0)	4.5 (4.4–4.7)	4.8 (4.5–5.0)	4.8 (4.6–5.0)	4.8 (4.6–4.9)

**Figure 3.**
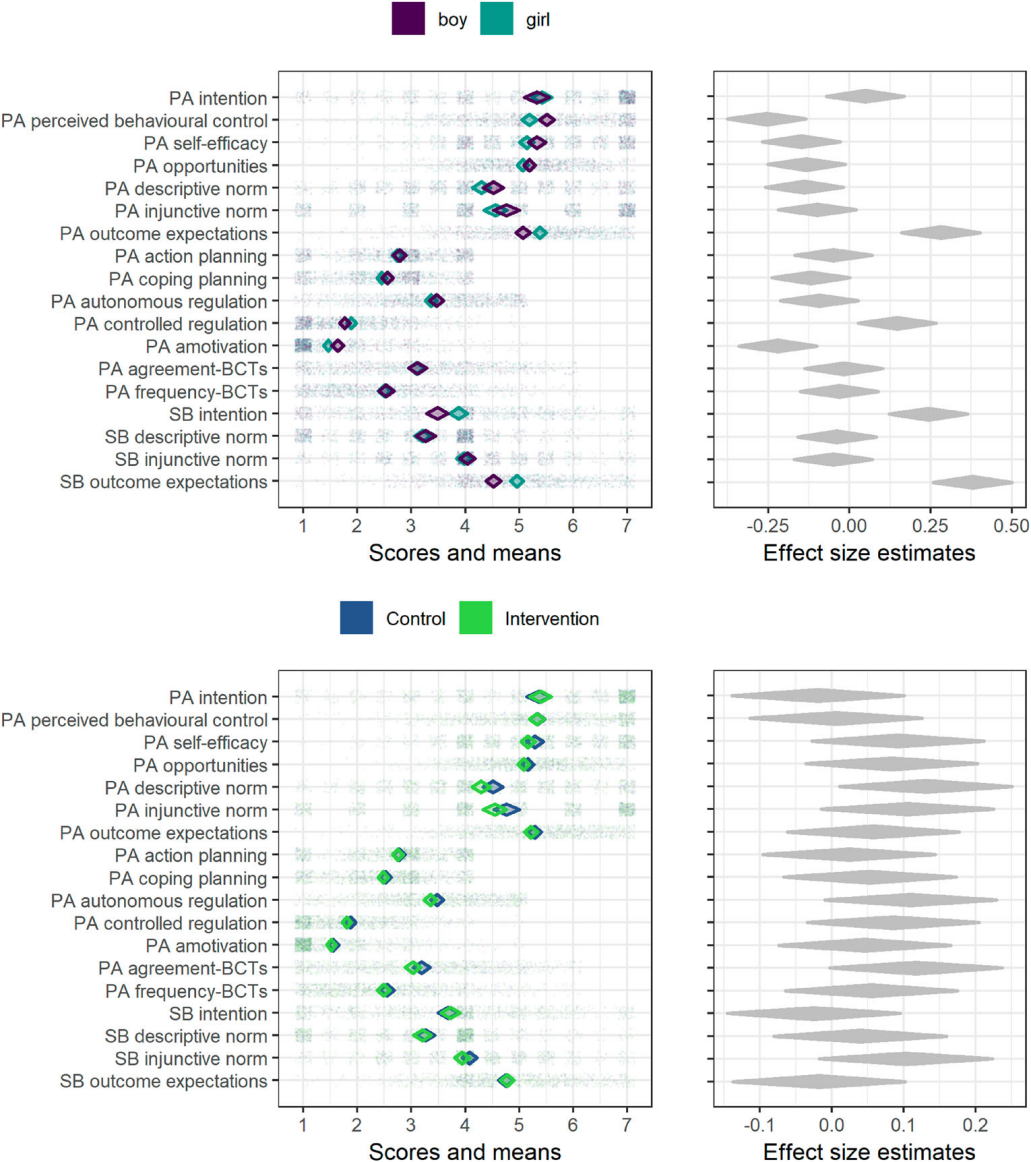
Diamond comparison plot drawn with R package ufs (Peters (2019), code available at https://git.io/fjLBB), showing means (middle of diamonds), 99% confidence intervals (endpoints of diamonds) and individual answers (dots) separated by gender and arm. Rightmost plots show heuristic effect sizes for differences in means (transformed to Pearson’s r). ICC is not accounted for in any plot.

In 14 of the 18 variables presented here, the mean of the control group is more favourable than that of the intervention group (average unadjusted advantage 1.91%). In Figure 3, the results are visualised in a concise manner.

From the left side of Figure 3, we can e.g. observe, that SB descriptive norms are bimodal (observations are clustered in answer options 1 and 4) and thus the means are not representative of typical participants. In addition, several of the variables are skewed (e.g. PA intention and PA amotivation), which has implications on analytical choices as well as interpretations of the mean values. On the right side, the effect size estimates indicate highest difference between genders in SB outcome expectations, and highest difference between treatment arms in PA descriptive norms – the overlap, though, is large and likely underestimated due to not taking cluster memberships into account (see methods).

#### Behaviour change technique usage

**Figure 4.**
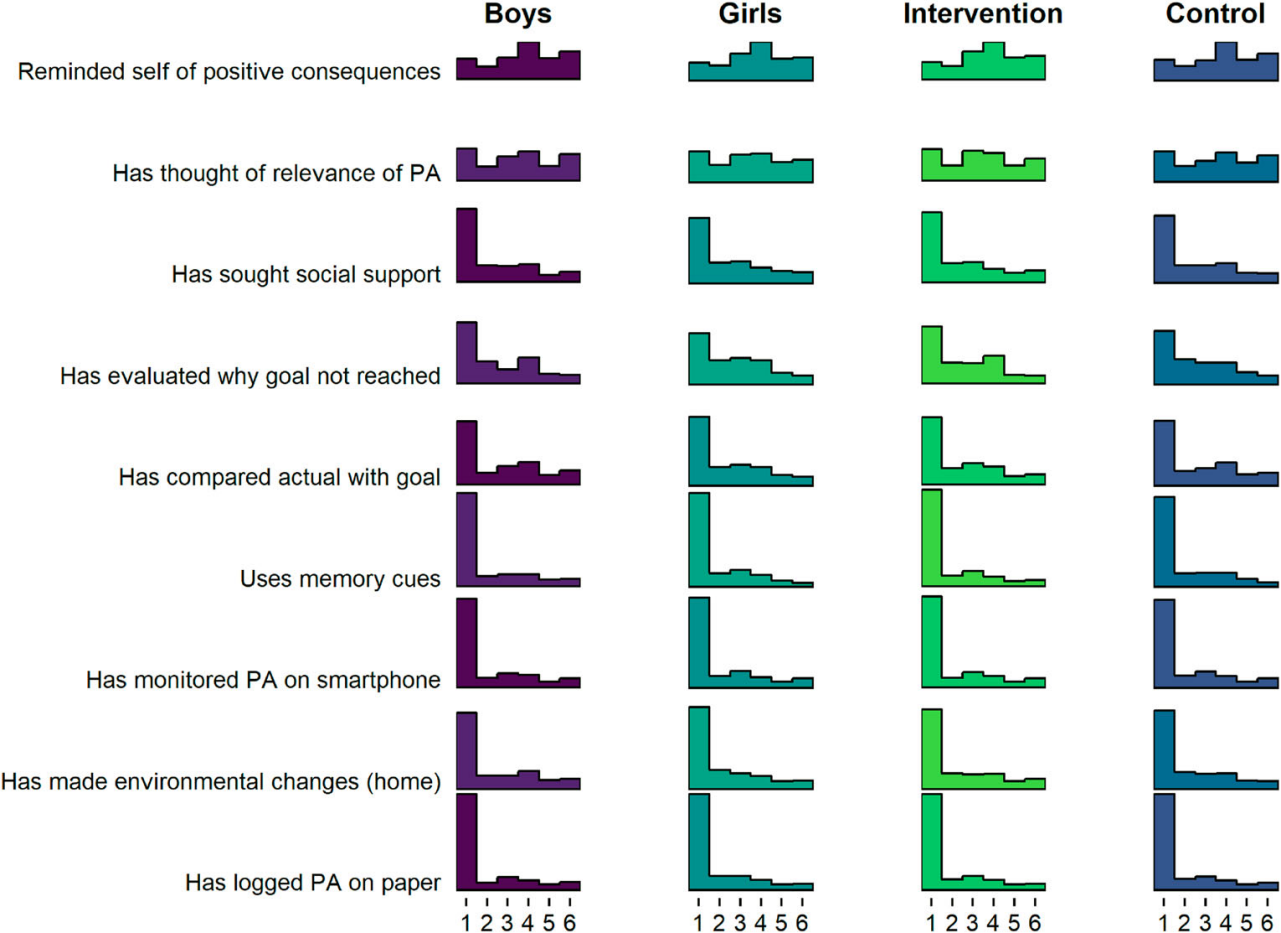
Histogram drawn with R package ggridges (Wilke and ggridges (2018), code available at https://git.io/fpOLj), showing self-reported use of frequency-dependent BCTs (1 = Not once … 6 = Daily).

There were no clear differences in frequency-dependent BCT use between genders or arms (Figure 4).

Figure 4 tells that the most frequent response is 1, indicating non-use of that BCT. In fact, a large number of BCTs seem to indicate a composite distribution, where one population reports never using the BCT, and another is seems normally distributed around the middle of the scale.

**Figure 5.**
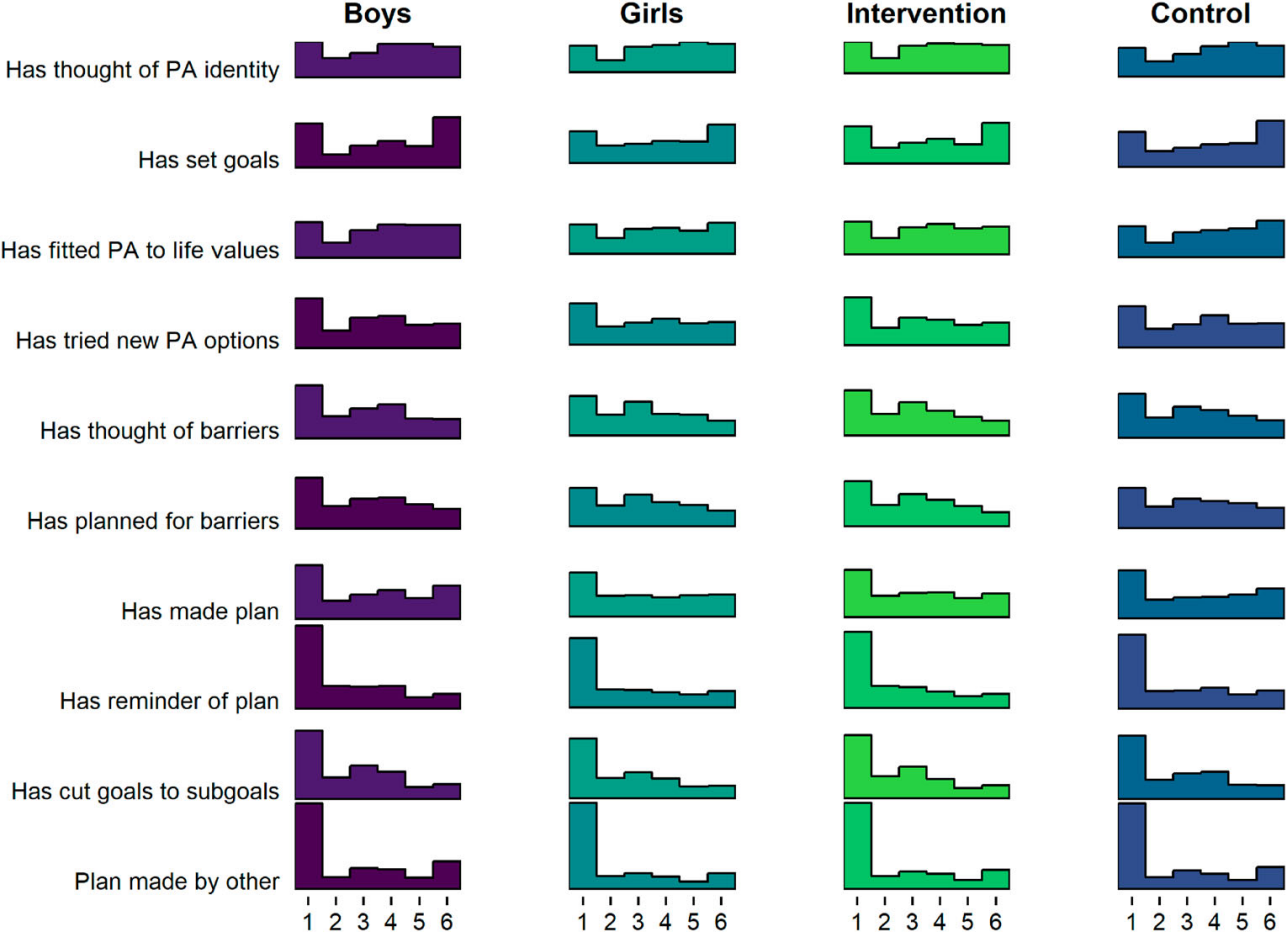
Histogram drawn with R package ggridges (Wilke and ggridges (2018), code available at https://git.io/fjLBE), showing self-reported use of agreement-dependent BCTs (1 = Not at all true … 6 = Completely true).

The aforementioned forms can also be observed in the distributions of agreement-dependent BCTs, as presented in Figure 5.

### Demonstration of network analysis

**Figure 6.**
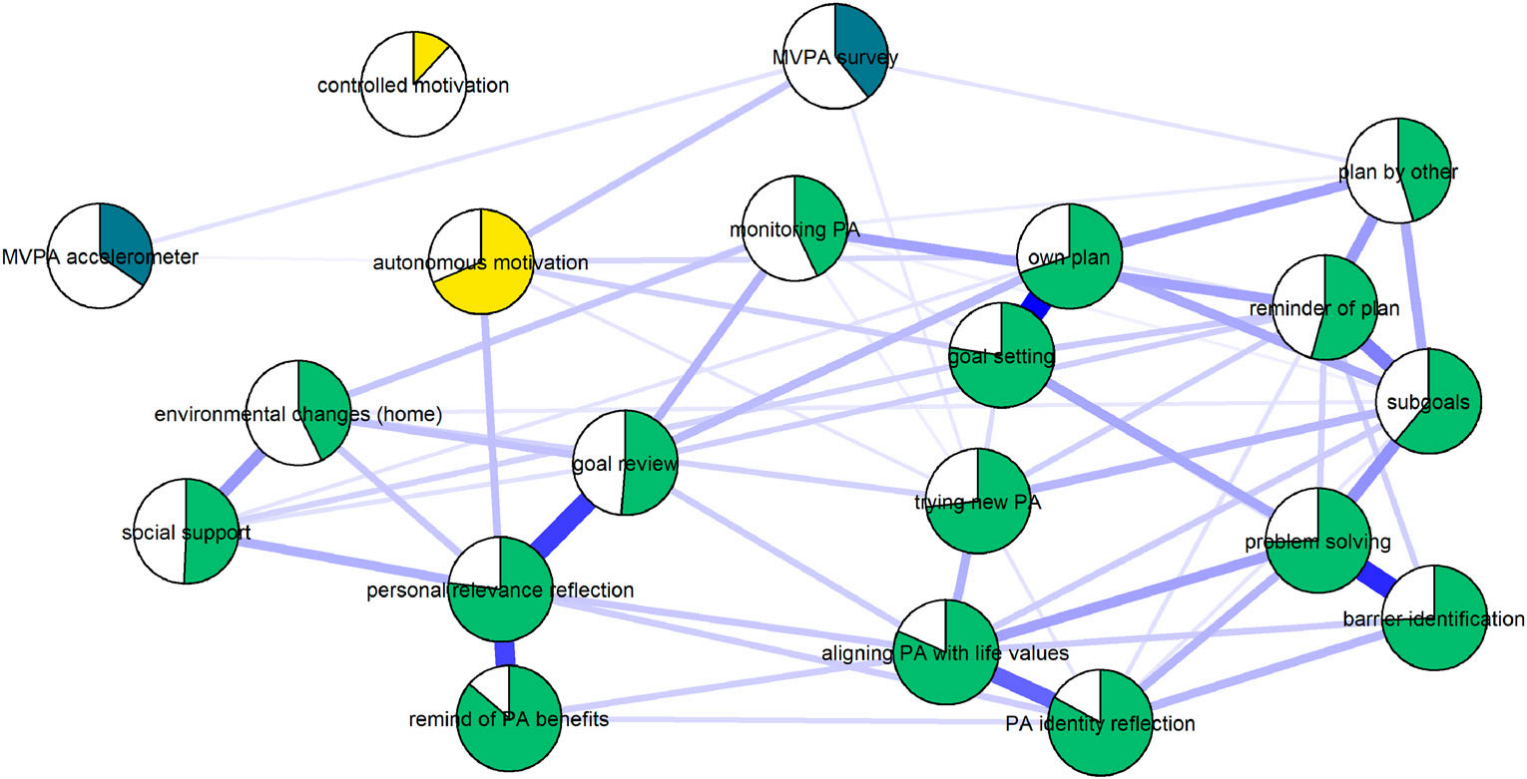
Mixed graphical model with LASSO regularisation and model selection by EBIC. Network models estimated and drawn with packages mgm (Haslbeck, 2019) and qgraph (Epskamp et al. (2019), code available at https://git.io/fpOXV). Blue lines indicate positive relationships. Plot shows the conditional dependence relationships between the variables of interest (edges which connect nodes), which can be interpreted akin to partial correlations. Pies depict means as proportion of theoretical maximum (in the case of accelerometer-measured moderate-to-vigorous physical activity (MVPA), mean as proportion of highest observed value); behaviour change technique (BCT) use and controlled motivation are dichotomised (see Methods). Node colours distinguish the three types of nodes; MPVA (blue), motivation (yellow), and BCT use (green).

Figure 6 shows a LASSO regularised mixed graphical model of BCT use, motivation and the two MVPA measures. We can observe, that after taking into account all the other nodes in the network and regularising small connections to zero, autonomous motivation appears to serve as a link between many BCTs and MVPA. In fact, only having a plan made by someone else, and having tried out new ways to be physically active (during the past three weeks), are directly connected to either of the MVPA nodes. In addition, use of certain BCTs are coupled particularly closely: Comparatively strong links exist between goal setting and having an own PA plan, between identifying barriers and planning to overcome them (i.e. problem solving/coping planning), and between goal setting and an own PA plan (i.e. action planning). We can also see a triad, where reflecting positive consequences is connected to goal review, through having thought of personal reasons to do PA, as well as less strongly coupled social support and having made changes to home environment. Such connections can be understood as variables influencing each other, but can also be indicative of underlying latent variables (i.e. the three variables are causal consequences of a shared origin) (Molenaar, 2010).

## Conclusions

This study investigated the baseline characteristics of the Let’s Move It trial cohort, making use of modern tools to visualise key results and exhaustively report the analyses, findings and analytical choices made. We found high levels of sedentary behaviour in the sample, with heterogeneity across educational tracks. MVPA, motivation and BCT use were modelled as a network, which highlighted the relevance of autonomous motivation in associations between PA and BCT use.

In contrast to earlier international and Finnish data collected in the general population (e.g. Husu, Suni, et al. (2016)), girls performed slightly more PA than boys in this sample. This is due to the practical nurse track being most active and mostly female; in other words, after accounting for track, no meaningful gender differences in accelerometer-measured MVPA could be seen. Further, boys reported doing more MVPA than girls, and the accelerometer-measurement implied boys were also more sedentary and interrupted sitting less often. Intervention and control groups were similar in their accelerometer-measured MVPA. This observation supports the decision of pairing educational tracks in randomisation, such that all tracks were represented in both arms. The practical nurse track was simultaneously the largest, the most active and had the highest percentage of girls, which means that potential gender differences in eventual intervention results should be interpreted with caution.

To our knowledge, this is one of the first studies to measure the use of potential BCTs comprehensively already at the trial baseline. As can be expected, many people indeed do use BCTs even before the intervention takes place. The results reveal that in the past three weeks, many participants report not having used self-regulation related BCTs such as planning, problem solving or goal setting, which on the other hand have been indicated to be useful techniques for PA self-management (Michie, Abraham, Whittington, McAteer, & Gupta, 2009). To our knowledge, this is also the first trial to measure the use of a range of BCTs among both control and intervention arm participants.

Comprehensive, transparent reporting of results leads to a vast amount of information to be presented: visual exposition is thus vital. Visualising distributions makes the variability among study participants more salient, which informs us about the distributional assumptions that underlie many common statistical techniques. Modern and traditional approaches to data visualisation also allow us to go further than just comparing means (Rousselet, Pernet, & Wilcox, 2017), and provide opportunities to avoid drawing false conclusions (e.g. in the case of Simpson’s paradox) based on summary statistics alone.

The results of the network analysis highlight, how most naturally used BCTs – exceptions including having a plan made by someone else, and trying out new forms of PA – possibly require autonomous motivation to affect MVPA. This finding, if corroborated in longitudinal data, would support the theoretical framework of the intervention, which held autonomy support and behavioural experiments at the forefront. So far, network models have been largely used as a tool for exploring empirical relationships among variables, often with little existing theory (Fried et al., 2017; Mõttus & Allerhand, 2017). One could understand this as the first generation of network papers in psychology, and there have been recent calls for a second generation that is confirmatory in nature, and based on existing theories of relationships among biological, psychological and social variables (Fried & Cramer, 2017).

The study also has limitations. It should be noted that while we consider 7-day accelerometry (with inclusion criterion of accumulating more than 4 days of over 10 h wear time) an approximation of a participant’s true habitual PA and SB in their daily life, it is not an errorless measure and it does not capture all forms of activity. Additionally, the questionnaire to measure the BCTs requires future validation (Bringmann & Eronen, 2016; Flake & Fried, 2019; Hankonen, 2018).

In the network model used, regularisation techniques are applied to remove spurious relations and control for multiple testing (for an in-depth tutorial on such regularised network models, see Epskamp and Fried (2018), and for a health psychology specific use case, see Hevey (2018)). At the same time, these networks estimate relations that are akin to partial correlations to derive the conditional dependence structure among variables. Potential pitfalls of these models and their application have been discussed elsewhere in detail (Fried & Cramer, 2017; Guloksuz, Pries, & Van Os, 2017). Most importantly, while in social networks one can include all relevant nodes (e.g. all people in a classroom or company), this is not so in biopsychosocial networks, where the question of what items to include as nodes remains a challenging question. Relations among items are often interpreted as putative causal pathways (although many other interpretations exist, Epskamp and Fried (2018)), which means one should not include two variables that are simply two indicators of the same construct (e.g. the items ‘I often feel sad’ and ‘I often feel blue’). Another important challenge is that one should avoid statistically controlling for common effects, also known as colliders: If in the true model both A and B independently cause C, C is a collider. If one controls for C in the model, a negative relation between A and B will emerge where no relation exists in the true model. This applies to all regression models and network models that are based on regressions, and it can be challenging to determine if a given variable is a collider. Rohrer (2018) provides an approachable introduction to causal inference in observational data.

The type of supplement used for this manuscript allows for presenting a lot, but not all, information due to resource considerations. One of the reader groups not fully considered are researchers and educators, who wish to use these data to guide intervention design. We would like to point out that the results, like most of the research in the area, only provide a group-level snapshot of a wide variety of constantly unfolding dynamic processes. Few individual participants are described by the group-level summary statistics: In fact, using Daniels’ (Daniels, 1952) definition of an ‘approximately average individual’ as falling in the middle 30% of the range of values, only 1.50% of participants can be considered ‘average’ on all of the primary outcome measures (see supplementary website, section https://git.io/fpOy1). Intervention designers looking at this cohort to choose to-be-targeted determinants for their study may want to consider applying clustering techniques on the data once it becomes publicly available. Still, and especially when processes are considered, group-level data does not inform the individual-level mechanisms of action in the case of non-ergodic systems, and hence the agreement between features of these two levels should be investigated (Fisher, Medaglia, & Jeronimus, 2018).

In conclusion, this analysis of baseline data from the Let’s Move It intervention trial indicates that randomisation did not result in highly disproportionate groups, i.e. the differences between arms were small – although, in the case of complex systems, even minimal differences may proliferate and lead to group imbalances (Rickles, 2009). It also highlights that vocational school students differ in many regards by their chosen educational track. Finally, graphical methods of presenting descriptive data are an important addition to traditional tables displaying means and standard deviations, which are most informational for symmetric distributions. Conventional approaches would have e.g. left the reader with an impression that the means of the multimodal or skewed variables are interpretable as central tendencies, and that the sample is homogenous. Transparent and accessible sharing of data characteristics, analyses and analytical choices is imperative for increasing confidence in research findings.

In the past, adopting methods such as the ones presented here, have come with large barriers to entry. Nowadays, with increased access to learning resources (such as code.org, khanacademy.org or datacamp.com), the increased appreciation of coding (Bers, 2017), as well as technology’s rising role in minimising research errors (Rouder, Haaf, & Snyder, 2018) and facilitating collaboration (Pain, 2018), these barriers are being torn down. Hence, we are confident that approaches such as this will become easier to adopt for the research community in the coming years. In high-quality RCTs with pre-specified outcomes, the exploratory data analysis techniques presented here have a role in detecting unintended effects commonly observed in complex systems (Moore et al., 2019). In such trials, the graphical representation of data retains its importance in conveying information, which promotes non-dichotomous thinking about statistical significance tests or confidence intervals (Amrhein, Greenland, & McShane, 2019; Mayo, 2018, p. 10), and elaborate supplements can act as a platform to present robustness tests and assumption explorations in.

## Abbreviations

**Abbreviations:** BA: Business and administration; BCT: Behaviour change technique; HRC: Hotel, restaurant and catering studies; IT: Business information technology; MVPA: Moderate-to-vigorous physical activity; Nur: Practical nurse; PA: Physical activity; SB: Sedentary behaviour

## Prior versions

A pre-print has been uploaded to PsyArxiv (DOI: 10.31234/osf.io/46rzm).

## Preregistration

Trial Registration Number: ISRCTN10979479. Registered retrospectively: 31.12.2015.

## Data, materials, and online resources

The analysis data will be available at https://osf.io/jn9ax/ after the anonymisation process has been completed by the end of 2019. All analyses and code are available at https://git.io/fNHuf (permalink at Heino and Sund (2019), GitHub repository at https://git.io/fjIQ6). The electronic questionnaire form is available at https://git.io/fjIP5.

## Reporting

We report all data exclusions, all manipulations, and all measures in the study. Sample size determination is reported in Hankonen et al. (2016).
